# Molecular Mechanism of Thymidylate Synthase Inhibition by N^4^-Hydroxy-dCMP in View of Spectrophotometric and Crystallographic Studies

**DOI:** 10.3390/ijms22094758

**Published:** 2021-04-30

**Authors:** Piotr Maj, Adam Jarmuła, Piotr Wilk, Małgorzata Prokopowicz, Wojciech Rypniewski, Zbigniew Zieliński, Anna Dowierciał, Agnieszka Bzowska, Wojciech Rode

**Affiliations:** 1 Nencki Institute of Experimental Biology, Polish Academy of Sciences, 3 Pasteur St., PL-02-093 Warszawa, Poland; a.jarmula@nencki.edu.pl (A.J.); z.zielinski@nencki.edu.pl (Z.Z.); anna.dowiercial@gmail.com (A.D.); 2 Helmholtz-Zentrum Berlin, Macromolecular Crystallography (NP-GMX) Elektronenspeicherring BESSY II, 15 Albert-Einstein-St., D-12489 Berlin, Germany; wilk.piotr@uj.edu.pl; 3 College of Inter-Faculty Individual Studies in Mathematics and Natural Sciences and Faculty of Physics, University of Warsaw, PL-02-097 Warszawa, Poland; malgorzata.prokopowicz@student.uw.edu.pl; 4 Institute of Bioorganic Chemistry, Polish Academy of Sciences, PL- 61-704 Poznań, Poland; wojtekr@ibch.poznan.pl; 5 Division of Biophysics, Institute of Experimental Physics, Faculty of Physics, University of Warsaw, PL-02-093 Warszawa, Poland; Agnieszka.Bzowska@fuw.edu.pl

**Keywords:** thymidylate synthase, dihydrofolate production, N^4^-hydroxy-dCMP binding, imidazolidine ring opening

## Abstract

Novel evidence is presented allowing further clarification of the mechanism of the slow-binding thymidylate synthase (TS) inhibition by N^4^-hydroxy-dCMP (N^4^-OH-dCMP). Spectrophotometric monitoring documented time- and temperature-, and N^4^-OH-dCMP-dependent TS-catalyzed dihydrofolate production, accompanying the mouse enzyme incubation with N^4^-OH-dCMP and N^5,10^-methylenetetrahydrofolate, known to inactivate the enzyme by the covalent binding of the inhibitor, suggesting the demonstrated reaction to be uncoupled from the pyrimidine C(5) methylation. The latter was in accord with the hypothesis based on the previously presented structure of mouse TS (cf. PDB ID: 4EZ8), and with conclusions based on the present structure of the parasitic nematode *Trichinella spiralis*, both co-crystallized with N^4^-OH-dCMP and N^5,10^-methylenetetrahdrofolate. The crystal structure of the mouse TS-N^4^-OH-dCMP complex soaked with N^5,10^-methylenetetrahydrofolate revealed the reaction to run via a unique imidazolidine ring opening, leaving the one-carbon group bound to the N(10) atom, thus too distant from the pyrimidine C(5) atom to enable the electrophilic attack and methylene group transfer.

## 1. Introduction

Thymidylate synthase (EC 2.1.1.45) catalyzes the dUMP methylation reaction, involving N^5,10^-methylenetetrahydrofolate (meTHF)-aided concerted transfer and reduction of the one-carbon group, its products being thymidylate and dihydrofolate (DHF) [1,2]. Several drugs used in anticancer, antiviral and antifungal chemotherapy are in their active forms either dUMP or N^5,10^-methylenetetrahydrofolate analogues inhibiting the enzyme [3,4,5].

**Scheme 1:**
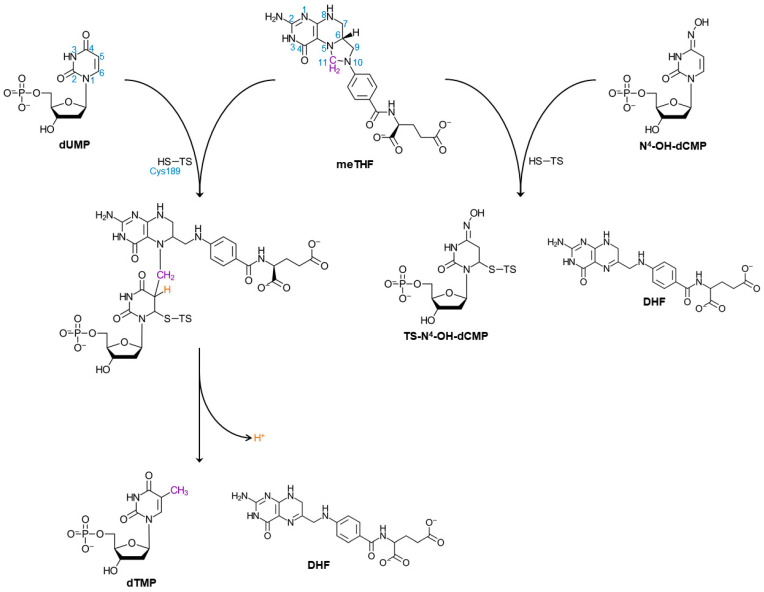
TS-catalyzed reaction (left) and the reaction between TS, N^4^-OH-dCMP and meTHF deduced from the crystal structure PDB ID: 4EZ8 (right); N^4^-OH-dCMP molecule depicted in the *anti*-imino form [9]. The meTHF one-carbon group is in purple, and atom numbering scheme and catalytic cysteine residue (Cys189 in mTS and *Tsp*TS but Cys197 in *Ce*TS) in blue.

N^4^-hydroxy-2′-deoxycytidine 5′-monophosphate (N^4^-OH-dCMP, Scheme 1) is a substrate analog, reported to inhibit the enzyme from bacterial, avian and mammalian sources, as well as from certain helminths. Inhibition was found to be competitive with respect to dUMP, and apparently mechanism-based, as it showed meTHF- and time-dependence; the dependence on time pointed to the slow-binding inhibition [6]. Although TS is a homodimer with two equivalent active sites, differing interactions were apparent between the low-molecular weight components (the inhibitor and meTHF) and each of the two eukaryotic enzyme subunits that may be interpreted in terms of negative cooperativity [7,8,9,10,11]. Similar to 5-fluoro-dUMP (FdUMP), N^4^-OH-dCMP incubated with the cofactor and the enzyme formed a ternary complex [8]. However, when N^4^-OH-[5-^3^H]dCMP replaced dUMP in the reaction mixture, ^3^H abstraction from the uracil ring C(5) was not apparent [9], suggesting the reaction to be inhibited at an earlier stage than with FdUMP, as the latter would arrest the reaction due to the presence of the fluorine, substituting C(5) hydrogen and incapable of dissociation [12,13].

Further crystallographic studies [14], resulting in mouse recombinant thymidylate synthase (mTS) crystal formed in the presence of N^4^-OH-dCMP and meTHF (PDB ID: 4EZ8), showed the enzyme involved in a ternary complex with the inhibitor and non-covalently bound product, DHF, instead of expected meTHF. This suggested the inhibition to result from an abortive enzyme-catalyzed reaction, involving a transfer of the one-carbon group to a hitherto unknown site and oxidation of THF to DHF. Moreover, the high resolution (1.17 Å) allowed us to conclude that both C(5) and C(6) inhibitor atoms exhibit the *sp*^3^ hybridization, suggesting C(5) reduction, with no apparent proton release from C(5). The active site in the mTS-N^4^-OH-dCMP-DHF structure was closed, the catalytic Cys189 being covalently bound to the C(6) atom of N^4^-OH-dCMP, reflected by the continuous electron density and distance of 1.87 Å between the C(6) and Cys189 Sγ sulfur atom (Scheme 1). In accord, N^4^-OH-dCMP remained bound to the enzyme under conditions of the SDS electrophoresis [15]. Of note is that in a ternary mTS complex, formed in the presence of FdUMP and meTHF (PDB ID: 5FCT; Table 1), all three components were covalently bound [13], as expected based on the corresponding TS structures published previously [12]. The latter proves meTHF to be stable under crystallization conditions, thus confirming that the previously mentioned structure (PDB ID: 4EZ8) is not an artifact.

In view of the foregoing, N^4^-OH-dCMP, similar to FdUMP, participates in an abortive enzyme-catalyzed reaction involving meTHF. However, while with FdUMP the reaction arrest follows the formation of the covalent ternary (enzyme-FdUMP-meTHF) complex, with the cofactor methylene group linking the pyrimidine C(5) and pteridine N(5), with N^4^-OH-dCMP the one-carbon group is transferred to a hitherto unknown site and THF becomes oxidized to DHF with associated pyrimidine C(5) reduction. As a result, the pyrimidine C(5) is not substituted by methyl, but acquires an additional hydrogen atom (Scheme 1). In order to test the above hypothesis, in the present study DHF production was followed accompanying TS incubation with N^4^-OH-dCMP in the presence of meTHF. Besides, structural studies were undertaken, testing (i) a possibility of TS of different specific origin, including the enzyme of the parasitic, *Trichinella spiralis*, and free-living, *Caenorhabditis elegans*, nematodes, to interact with the enzyme in a way similar to that showed by the mouse enzyme (PDB ID: 4EIN and 4EZ8) and (ii) the fate of meTHF in the reaction involving TS and N^4^-OH-dCMP.

The present results verify the TS-catalyzed DHF production, at a temperature-dependent rate, during incubation of the enzyme with N^4^-OH-dCMP and meTHF. Structural results provide further evidence that such incubation, also with the parasitic nematode *T. spiralis* enzyme, results in the inhibitor molecule being covalently bound at the active center via the pyrimidine C(6) and Cys189 sulfur, with the pyrimidine C(5) non-substituted by methyl, but acquiring an additional hydrogen atom. Further crystallographic results suggested the imidazolidine ring of meTHF, entering the binary TS-N^4^-OH-dCMP complex, to open on the N(5), instead N(10) side (cf. Scheme 1). The latter offers an explanation of inability of the methylene group, located on the N(10) and thus being too distant, to attack the inhibitor C(5).

## 2. Results

### 2.1. DHF Production in the Course of N^4^-OH-dCMP Incubation with TS and meTHF

**Figure 1:**
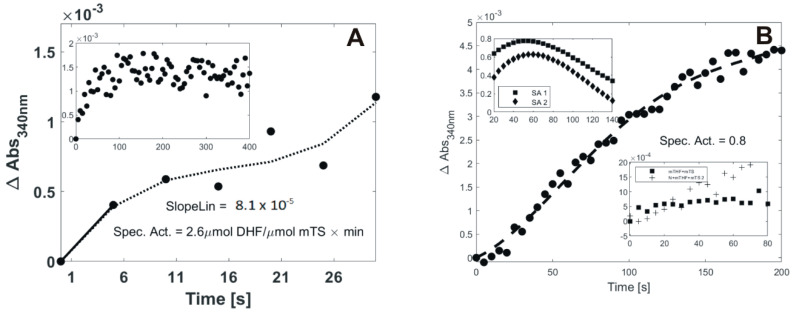
Time-dependent absorption change at 340 nm wavelength in the course of 5.0 µM N^4^-OH-dCMP and 5.0 µM meTHF incubation with 0.32 µM mTS (dimer) at 15 °C (**A**) or 0.40 µM mTS at 8 °C (**B**). (**A**) Absorption change at λ_obs_ = 340 nm during first 30 s of a measurement at 15 °C. The slope, indicative of the initial rate, was calculated based on the linear fit between t = 0 s and t = 5 s as SlopeLin = 8.1 × 10^−5^. Inset: ΔAbs in a range of 0 to 400 s (λ_obs_ = 340 nm). SlopeLin-slope of the linear fit between t = 0 s and t = 5 s. Spec. Act. is “specific activity” calculated as (SlopeLin × *f*): ([mTS] × 2 × 10^−4^) × 60, where *f* (calculated as 10,000: 58 × 2 × 10^−4^) is the correction factor allowing the results to be expressed in units of DHF µmoles per second in a sample and [mTS] is the concentration of the enzyme dimer expressed as µM. (**B**) Absorption change at λ_obs_ = 340 nm in the 0 to 200 s time range at 8 °C. Upper Inset: values of the Specific Activities (SA1, SA2) plotted vs. time. SA1 and SA2 were calculated based on the tangents at each data point on each of the two curves reflecting one of the two measurement repeats. Lower inset-exemplary data of the control [meTHF + mTS (+)] and full [N^4^-OH-dCMP + mTHF + mTS (■)] reactions in the 0 to 80 s time range. To calculate the “specific activity” (Spec. Act.), an interpolation function was calculated to the data points. Subsequently, tangents to each point of the fitted curve were computed, and the maximum tangent’s value chosen from the data range with comparable tangents values.

In order to verify DHF as a product of the N^4^-OH-dCMP-dependent mTS interaction with meTHF, time-dependent absorption increase at ʎ_obs_ = 340 nm was observed, accompanying the enzyme incubation with the inhibitor and cofactor (Figure 1). Notably, the control reaction, involving meTHF incubated with the enzyme and monitored under the same conditions, showed no apparent time-dependent absorption increase.

While the results obtained at 15 °C (Figure 1A) clearly pointed to DHF production, the inset shows the increase to be registered only within a short time range (0 to ca. 30 s), with a visible diminution of the absorption change per time interval, eventually reaching a plateau after 60 s. Hence, in order to capture a linear part of the plot reflecting ΔAbs vs. time relationship, the slope of the linear fit between t = 0 s and t = 5 s was calculated, and used to assess the enzyme specific activity (SA) value (2.6 µmol DHF produced by 1 µmol mTS per 1 min for the experiment presented in Figure 1A, and the mean value calculated for *n* = 4 experiments with indicated standard error being 2.6 ± 0.2 µmol DHF produced by 1 µmol mTS per 1 min). At lower temperature (8 °C, Figure 1B) the reaction was slowed down, with the absorption increase rate reaching a plateau after around 200 s. It should be noted that negative values at the start of the reaction (Figure 1B, the lower insert) resulted from an instant absorption increase (within the initial 5–10 sec), faster in the control (meTHF + mTS) than full (N^4^-OH-dCMP + meTHF + mTS) reaction (cf. the lower inset), due presumably to a difference in temperature equilibration between the control and full reaction samples. In the upper inset, changes with time in the course of the reaction are presented of the specific activity values, based on the tangents values reflecting slope changes along the fitted curve corresponding to the corrected absorption increase with time. For both samples, acceleration of the reaction was observed until ca. 50–60 s; then it reaches a maximum velocity, followed by its decline. The specific activity, reflecting an apparent initial reaction rate, was calculated for the experiment presented in Figure 1B as 1.5 µmol DHF produced by 1 µmol mTS per 1 min (the mean value calculated for *n* = 3 experiments with indicated standard error was 1.2 ± 0.3 µmol DHF produced by 1 µmol mTS per 1 min).

### 2.2. Crystallographic Studies

In order to learn more about the mechanism of TS inhibition by N^4^-OH-dCMP, the following crystallographic experiments were carried out: (i) co-crystallization of *Tsp*TS with N^4^-OH-dCMP and meTHF, i.e., an experimental set analogous to the one that resulted in the previously mentioned mTS crystal (PDB ID: 4EZ8 [14]), (ii) co-crystallization of *Ce*TS with N^4^-OH-dCMP (PDB ID: 4PSG) and (iii) soaking with meTHF solution of a crystal of the binary complex mTS-N^4^-OH-dCMP [14], resulting from mTS co-crystallization with N^4^-OH-dCMP (PDB ID: 4EIN). Further description of the crystal structures obtained is presented below. Of note is that the three resulting structures, similar to those of the previously studied TS-N^4^-OH-dCMP complexes (PDB IDs: 4EZ8 and 4EIN [14]), showed the inhibitor molecule to be present in the *anti*-imino form.

### 2.3. Crystal Structure of CeTS Soaked with N^4^-OH-dCMP (PDB ID: 4PSG)

**Figure 2:**
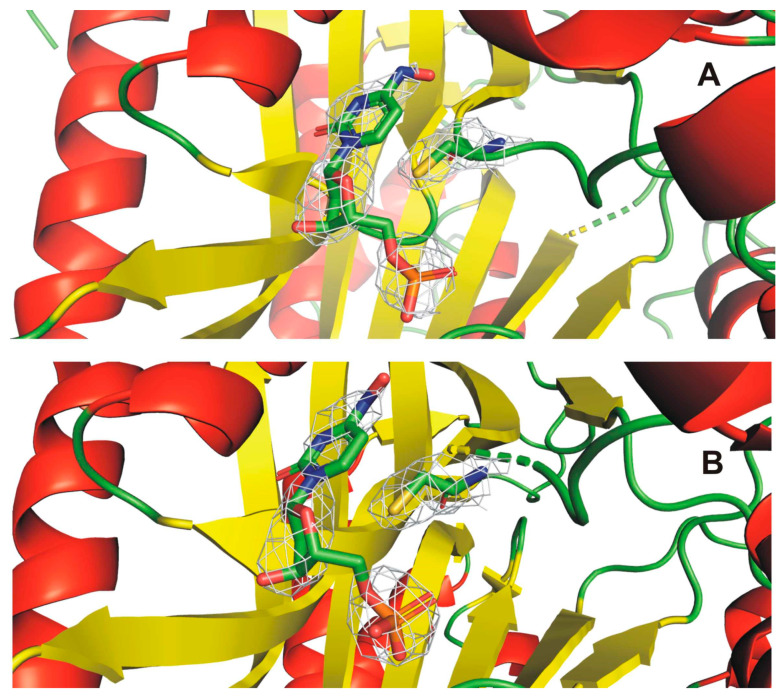
Active sites of *Ce*TS visible in the enzyme crystal soaked with N^4^-OH-dCMP (PDB ID: 4PSG). Panels (**A**,**B**)—subunits A and B, respectively. OMIT electron density maps contoured at 2σ for N^4^-OH-dCMP and Cys197.

Attempts were unsuccessful to crystallize a binary *Tsp*TS-N^4^-OH-dCMP complex, preventing a contemplated comparison of its structure with that of the ternary complex formed by *Tsp*TS with N^4^-OH-dCMP in the presence of meTHF. However, the structure of the corresponding binary complex with *Ce*TS substituting *Tsp*TS, and modeling the nematode enzyme, was successfully obtained (PDB ID: 4PSG). It presents a single *Ce*TS dimer in the asymmetric unit and allows resolution of 2.8 Å, thus limiting structural information that can be deduced from the structure. Residues 24-312 of both subunits were modeled, with N^4^-OH-dCMP molecules in both active sites refined with full occupancy (Figure 2). Considering a relatively low resolution of the 4PSG structure, the average B-factor values are surprisingly low for subunits A and B, and for the whole structure, amounting to 16.3Ǻ^2^, 16.9Ǻ^2^ and 16.6Ǻ^2^, respectively. The two subunits A and B are also highly similar to each other in terms of the overall structure, with a Cα RMSD value between them equal to 0.32 Å, as calculated with MUSTANG [16]. The N^4^-OH-dCMP molecules are situated far enough from the corresponding catalytic cysteine residues (Cys197 in *Ce*TS) to exclude the possibility of existence of covalent bonds (the distances are 3.78 Å and 3.60 Å in subunits A and B, respectively).

### 2.4. Crystal Structure of TspTS Cocrystallized with N^4^-OH-dCMP and meTHF (PDB ID:5M4Z)

**Figure 3:**
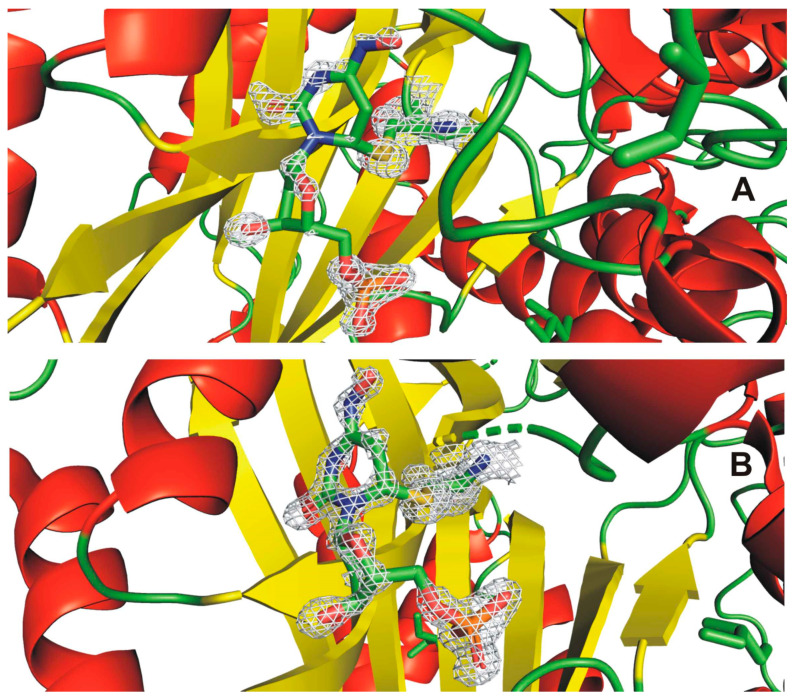
Active sites of *Tsp*TS co-crystallized with N^4^-OH-dCMP and meTHF (PDB ID: 5M4Z). Panels (**A**,**B**)—subunits A and B, respectively. OMIT electron density maps contoured at 2σ for N^4^-OH-dCMP and Cys189.

An asymmetric unit contains a single *Tsp*TS dimer. Residues 18–41 and 45–307 of chain A and 18–303 of chain B were modeled. Within each of the two active sites a clear electron density was present, identified as the N^4^-OH-dCMP molecule (Figure 3). However, no electron density was found that could be unequivocally identified as a folate derivative molecule. Both N^4^-OH-dCMP molecules were refined with high occupancy (0.95 and 0.93 in subunits A and B, respectively), with each N^4^-OH-dCMP pyrimidine ring conformation pointing to *sp*^3^ hybridizations of the C(5) and C(6) atoms, and the distance between pyrimidine C(6) and Sγ of Cys189 suggesting a covalent bond to be present between N^4^-OH-dCMP and each of TspTS subunits.

Both TS subunits of the discussed structure are very similar, as reflected by the Cα RMSD between them amounting to 0.74 Å, with most differences present at the C-terminal part of the protein. The C-termini of the subunits A and B are in the open and closed conformations, respectively (the last four residues in subunit B were not modeled due to poor and ambiguous electron density).

**Figure 4:**
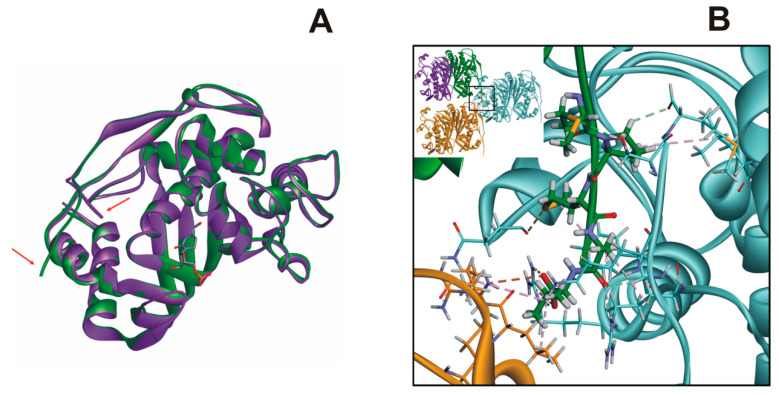
C-termini of *Tsp*TS co-crystallized with N^4^-OH-dCMP and meTHF (PDB ID:5M4Z). (**A**) Superimposed subunits A and B (green and purple, respectively) with C-termini marked by red arrows. (**B**) Depiction of crystal contacts between the final four C-terminal residues from subunit A (green carbon atoms and cartoon, and thick stick representation) and relevant symmetry related TS dimers (light blue or orange carbon atoms and cartoon, and thin stick representation). Inset: relative spatial orientation of depicted TS dimers.

Electron density maps fully support the way the C-termini of this structure were modeled and their configuration can be at least partially explained. The C-terminus of subunit A is stabilized by crystal contacts with several residues from the neighboring TS dimer (Figure 4). The lack of clearly defined electron density for the final few (four in this case) residues of the C-terminus of subunit B has been observed previously for several structures of TS complexes, e.g., rat TS-dUMP-Tomudex (PDB ID: 1RTS and 2TSR [17]), *Ce*TS-dUMP-Tomudex (PDB ID: 5NOO [18]), and mTS-dUMP-Tomudex (PDB ID: 4EB4 [19]).

Temperature factors B of this structure are relatively low, with slightly higher values for the subunit B than A (averages of 26.3 Å^2^ and 20.7 Å^2^, respectively) and the total average value (without water molecules) of 23.5 Å^2^. High B-factor values were observed specifically for the loop 41–47 of subunit B. High mobility of this loop is somewhat surprising as it includes Arg43, one of the four arginine residues anchoring the nucleotide’s phosphate group with hydrogen bonds to its oxygens.

The lack of the folate derivative, presumably DHF, constitutes the most glaring difference between this structure and the aforementioned analogous mTS structure (PDB ID: 4EZ8). Although in the subunit A it may be attributed to the open conformation of the C-terminal loop, closed conformation of C-terminus of subunit B is characteristic for ternary complexes in which C-terminal residues are involved in non-bonding interactions with the cofactor or its analogue.

DHF is most likely the folate derivate that was released from the structure, based not only on the same ligands used for crystallization with both 4EZ8 and 5M4Z structures but also on the same changes that occurred to the inhibitor molecules in both structures. They amount to the pyrimidine ring C(5) atom reduction, of both pyrimidine C(5) and C(6) *sp*^3^ hybridization, as well as the covalent bond between C(6) and Sγ of TS Cys189.

**Figure 5:**
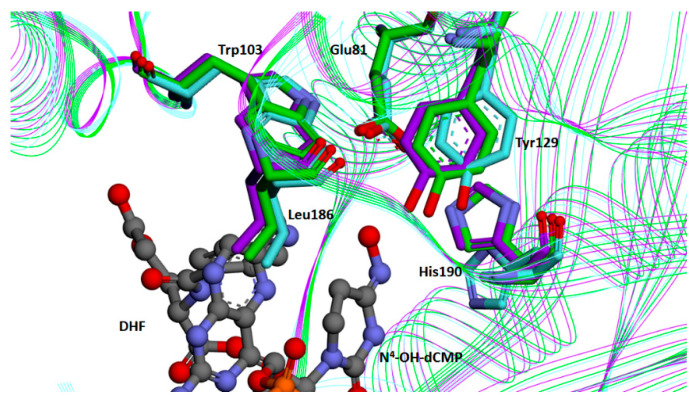
Amino acid residues in close proximity of N(4)-OH group of N^4^-OH-dCMP molecule bound in TS active site. Superimposed are subunits from structures 5M4Z (from this report) and 4EZ8 [14]. Subunits A and B from 5M4Z structure in green and purple, respectively; 4EZ8 in light blue. Carbon atoms of amino acid residues shown as sticks colored accordingly to their subunit. N^4^-OH-dCMP and DHF molecules from 4EZ8 structure are in ball-and-stick.

The residues Glu81, Trp103, Tyr129, Leu186 and His190 (numbering is the same for mTS and *Tsp*TS) are positioned relatively close to the N(4)-OH group of the inhibitor, therefore they can participate in non-covalent interactions with this group. Among them only residues Tyr129 and His190 are oriented differently in structures 4EZ8 and 5M4Z (Figure 5), with each of the two located farther from the N(4)-OH group in the mTS than the *Tsp*TS structure (Table 1). These differences correspond with the different distances between residues Tyr129 and His190 in both structures, although it does not seem to be affected directly by the respective absence/presence of DHF. In the 4EZ8 structure both residues are not close enough to interact with either DHF or other residues involved in such interactions.

Spatial orientation of His190 present in the 4EZ8 structure (or of the corresponding histidine residue in any other specific variant of TS) is also found in multiple other structures of the mouse enzyme, e.g., 4EIN (mTS-N^4^-OH-dCMP complex [14]), 4E5O (mTS-dUMP [20]), 6F6Z (mTS-N^4^-OH-dCMP soaked with meTHF), 4EB4 (mTS-dUMP-Tomudex [19]), 3IHI (mTS apoenzyme [13]), and as one of two alternative conformations in 5BY6 (*Tsp*TS-dUMP [18,21]). On the other hand, an alternative orientation of His190 present in the 5M4Z structure is also observed in several other structures, including 5BY6 (as the other alternative conformation with 0.6 occupancy), 4PSG (*Ce*TS-N^4^-OH-dCMP complex), 5NOO (*Ce*TS-dUMP-Tomudex [18]), 5FCT (mTS-FdUMP-meTHF [13]), 4IRR (*Ce*TS-dUMP [19]), 4IQB (*Ce*TS apoenzyme [18]), and 4ISW (phosphorylated *Ce*TS-dUMP [22]). Therefore, it seems that those differences are stronger correlated with the TS sequence than with the occupation of the active site. The nematode TS structures (except for one of the 5BY6 conformations) have His190 (or His198 in the case of *Ce*TS) in similar conformation to the one present in the 5M4Z structure, whereas mTS structures (except 5FCT) are in that respect closer to 4EZ8.

### 2.5. Crystal Structure of mTS Cocrystallized with N^4^-OH-dCMP and Soaked with meTHF (PDB ID: 6F6Z)

An asymmetric unit contains a single mTS dimer. Residues 21–307 of both subunits were modeled. An average B-factor value’s distribution is well-balanced between the two subunits (48.8 Å^2^ and 50.9 Å^2^ for subunits A and B, respectively) and amounts to 49.9 Å^2^ for the whole structure (excluding water molecules). Subunits A and B are also similar in terms of the overall structure, with Cα RMSD value between them amounting to 0.22 Å.

**Figure 6:**
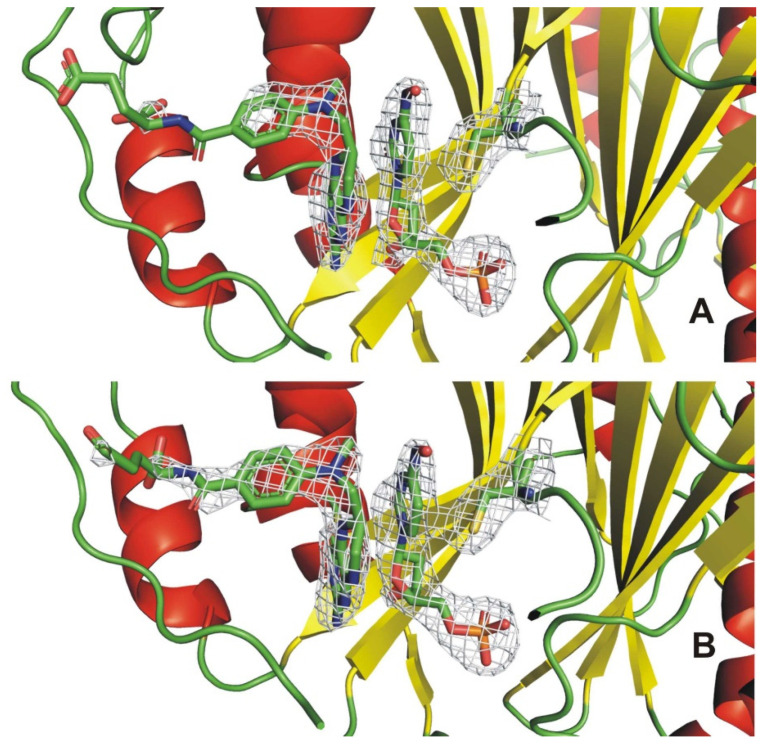
Active sites of mTS co-crystallized with N^4^-OH-dCMP and soaked in meTHF (PDB ID: 6F6Z). Panels (**A**,**B**)—subunits A and B, respectively. OMIT electron density maps contoured at 2σ for N^4^-OH-dCMP, folate derivative (PDB ligand code: TGQ) and Cys189.

Electron density corresponding to the inhibitor and the cofactor is clearly defined in both subunits (Figure 6). N^4^-OH-dCMP molecules are modeled with full occupancy. The folate derivative is modeled with 0.88 and 0.85 occupancies for subunits A and B, respectively. The N^4^-OH-dCMP molecules are situated too far away from Cys189 to form covalent bonds (the distances are 3.07 Å and 2.95 Å in subunits A and B, respectively).

In view of moderate resolution of 2.13 Å, identification of subtler structural features of ligands bound in the active site was not possible. In particular, the resolution is insufficient to unequivocally judge the oxidation levels of the pteridine ring and one-carbon group of the folate derivative (PDB ligand code: TGQ), even if scrutinized electron density maps allow leaning towards tetrahydrofolate (with the pteridine ring C(6) *sp^3^* hybridization) and methylene group (suggested as one resulting from the imidazolidine ring opening), respectively. Of note, however, is the fact that the cofactor molecule is present in a modified form, with electron density next to the N(10) atom, suggesting the one-carbon group to be covalently bound to N(10) instead N(5) (Figure 6). Thus the imidazolidine ring must have been apparently opened in an unusual manner, on the N(5) rather than N(10) side.

The 6F6Z structure does not allow a direct identification of factors responsible for the unusual opening of the imidazolidine ring, although the phenomenon appears related to the presence of the pyrimidine N(4)-OH substituent. Analysis of residues remaining in a close proximity to TGQ shows that the one-carbon group is orientated toward the inhibitor N(4) atom, separated from it by the distances of 3.43 Å and 3.34 Å in the subunits A and B, respectively. The corresponding distance to the N(4)-OH group oxygen atom is also similar: (3.72 Å and 3.47 Å in the subunits A and B, respectively). However, the N(4)-OH group is positioned farther from the pteridine N(5) than N(10) atom, the distances between the O(4) and N(5)/N(10) amounting to 4.99Ǻ/4.32Ǻ in the subunit A and 5.00Ǻ/4.24Ǻ in the subunit B.

Multiple experiments of the mTS-N^4^-OH-dCMP crystals soaking with a cofactor or its derivatives failed to yield new structures with noticeable electron densities corresponding to the soaking compounds, despite using the same crystallization conditions as those that led to the 6F6Z structure and testing multiple different soaking times and folate derivative concentrations.

## 3. Discussion and Conclusions

The present results allow learning certain new aspects of thymidylate synthase inhibition by N^4^-OH-dCMP. Spectroscopic studies were aimed at testing the hypothesis, based on the 4EZ8 structure, of DHF being produced in the reaction catalyzed by TS with N^4^-OH-dCMP and meTHF. The time- and temperature-, as well as N^4^-OH-dCMP-dependent DHF production in the course of N^4^-OH-dCMP incubation with TS and meTHF, reflected by absorption increase at λ_obs_ = 340 nm (Figure 1), verifies DHF to be released in the course of the N^4^-OH-dCMP-dependent mTS interaction with meTHF. Thus, as hypothesized, following removal, in a so-far unknown way, of the meTHF one-carbon group, the enzyme appears to use THF as a reducing agent in the absence of the pyrimidine C(5)=CH_2_ group. In accordance, evidence was recently demonstrated of the ability of TS to catalyze THF-dependent covalent binding of N^4^-OH-dCMP and FdUMP [23].

Crystallographic studies allowed a comparison of the structures of nematode (*T. spiralis* and *C. elegans*) TSs, 5M4Z and 4PSG, with those previously presented of mammalian (mouse) enzymes 4EZ8 and 4EIN, to assess structural consequences of the inhibitor interaction with the three TS specific variants differing by the aa sequences (for mTS and *Tsp*TS showing 67.4% identity and 87.7% similarity, and surprisingly only 60.5% identity and 85.5% similarity for the two nematode sequences [18]), in spite of the overall high evolutionary conservation of TS [24]. While previous studies showed N^4^-OH-dCMP to be a similarly potent slow-binding inhibitor of TS from different mammalian sources, as well as from certain parasitic helminthes, including the nematode *T. spiralis* [9,10], of interest was interaction of the inhibitor with the enzyme of *C. elegans*, considered a useful model for anthelminthic discovery [25]. Regarding the latter aspect, comparison of the binary, mTS-N^4^-OH-dCMP (4EIN) with *Ce*TS-N^4^-OH-dCMP (4PSG), as well as complexes formed in the presence of both N^4^-OH-dCMP and meTHF with mTS (4EZ8) or *Tsp*TS (5M4Z) could provide structural data that could be potentially useful in screening for species-selective anti-nematode inhibitors of the enzyme.

The lack of a folate derivative molecule in the *Tsp*TS-N^4^-OH-dCMP complex structure (PDB ID: 5M4Z) is something most glaringly differing it from the corresponding mTS ternary complex structure (PDB ID: 4EZ8). *Tsp*TS incubated with the inhibitor and meTHF might have catalyzed a reaction similar to that deduced to run in the mTS ternary complex and result in the 4EZ8 structure. If so, then with *Tsp*TS, unlike with mTS, the DHF molecule must have been released from the complex prior to crystallization. This claim is supported by the fact that N^4^-OH-dCMP molecules are present in both structures (4EZ8 and 5M4Z) in the same form (superimposed almost perfectly overlap), representing the product of a hypothetic abortive reaction, involving the pyrimidine ring C(5) reduction accompanied by THF oxidation to DHF without the usual one-carbon group transfer. The latter is reflected in both structures by the electron density maps indicating the *sp^3^* hybridization of each C(5) and C(6) atom of the pyrimidine ring, as well as the presence of a covalent bond between the Sγ atom of the active center catalytic cysteine residue and the C(6) atom, separated invariantly by an adequately short distance. On the other hand, structures obtained without meTHF present in the cocrystallized mixture, i.e., mTS-N^4^-OH-dCMP (PDB ID: 4EIN) and *Ce*TS-N^4^-OH-dCMP (PDB ID: 4PSG), as well as the structure of mTS cocrystallized with N^4^-OH-dCMP and soaked with meTHF solution (PDB ID: 6F6Z), are all revealing significantly larger distances between the C(6) of the inhibitor’s pyrimidine ring and the Sγ atom of the catalytic cysteine residue, rendering the formation of a covalent bond impossible. The said distances are equal to 2.85 Å and 2.93 Å in 4EIN structure, 3.78 Å and 3.60 Å in 4PSG structure, and 3.07 Å and 2.95 Å in 6F6Z structure (reported as values for subunits A and B from each crystal structure, respectively). The pyrimidine ring of each N^4^-OH-dCMP molecule present in those structures is flat, indicating *sp*^2^ hybridization of both the C(5) and C(6) atoms and an aromatic character of the pyrimidine ring. Apparently, the covalent bond formed between the N^4^-OH-dCMP molecule and the enzyme observed in both 4EZ8 and 5M4Z structures results from the reduction of the C(5) atom, accompanied by the loss of the aromatic character of the pyrimidine ring. The DHF molecule remaining non-covalently bound in the TS active site of the 4EZ8 crystal structure allowed us to identify this compound as a potential product of THF oxidization (THF is what remains after the methylene group release from meTHF, e.g., as formaldehyde [26]) of the abortive reaction occurring in the presence of N^4^-OH-dCMP. As for the 5M4Z structure, the lack of a folate derivative alongside covalently-bound N^4^-OH-dCMP molecule with reduced C(5) atom suggests that such a derivative (presumably DHF, the product of THF oxidization) was released to the crystallization solution.

No obvious reasons explain why DHF was released from the protein in the 5M4Z but remained non-covalently bound in the 4EZ8 crystal structure. One could speculate that the aforementioned differences in the conformation of C-terminal loops play a role in this phenomenon. The C-terminus open conformation in subunit A of the 5M4Z structure could explain the release of DHF but this hypothesis is not supported by the corresponding conformation of the subunit B being closed (note, however, that the C-terminus B was not fully modeled due to insufficient data). Comparison of aa residues involved in non-covalent interactions with the DHF molecule in the 4EZ8 structure with their counterparts in the 5M4Z structure reveals an interesting difference in orientation of the Leu215 sidechain. While this residue participates in hydrophobic interactions with DHF in the 4EZ8 structure, it is oriented in both subunits of the 5M4Z structure in a way that would weaken or even prevent such interactions. Other potential reasons involve differences in crystal packing, as the two structures belong to different space groups (C 2 2 2_1_ for 4EZ8 and P 1 for 5M4Z). It is also possible that applying different crystallization conditions would have affected DHF binding, resulting in DHF released from the *Tsp*TS complex. Finally, minor conformational differences between mTS and *Tsp*TS could lead to faster DHF release from the latter. Of note is that in the TS-catalyzed physiological reaction, DHF (in monoglutamate form) is the first product to be released [27]. Therefore, the state with DHF molecule present within the crystal structure (as in the 4EZ8 structure) seems to be an unusual but extremely fortunate occurrence from the point of view of our studies on N^4^-OH-dCMP inhibition mechanism.

Both available structures of TS-N^4^-OH-dCMP binary complexes obtained in the absence of meTHF, 4EIN and 4PSG, display a high level of similarity of enzyme-inhibitor interactions, even despite their different specific origin (mouse and *C. elegans*, respectively). Both contain N^4^-OH-dCMP molecules that are not bound covalently to TS catalytic cysteine residue (Cys189 in mTS, and Cys197 in *Ce*TS). Altered orientation of one of the histidine residues (His190 in mTS, and His198 in *Ce*TS) is the only noticeable difference in the active site of enzymes present in both structures. In the 4EIN structure, histidine is oriented closer to the N(4)-OH group, whereas in the 4PSG structure, it is turned away from the inhibitor and oriented more toward the Tyr137 ring.

A complex, resulting in the structure 6F6Z, formed by soaking mTS-N^4^-OH-dCMP with meTHF solution, was obtained in search of TS inactivation intermediate steps preceding that observed within the 4EZ8 structure (mTS ternary complex with the covalently bound inhibitor and non-covalently bound DHF; Scheme 1). A similar approach has proven successful in studies involving several groups of enzymes [28,29,30,31,32,33]. The N^4^-OH-dCMP molecule in the resulting complex is most likely not bound to the catalytic cysteine residue, thus being probably in the same conformation as the inhibitor molecule present in the binary mTS-N^4^-OH-dCMP complex structures (PDB ID: 4EIN and 4PSG). Of particular interest is the cofactor’s form (PDB ligand designation TGQ), a unique feature of the latter being the meTHF imidazolidine ring opening state, with the methylene group remaining bound to the N(10) atom. This is in contrast to the TS-catalyzed reaction with dUMP or FdUMP and meTHF when the imidazolidine ring opens at the N(10) site, and the methylene group linking the meTHF N(5) and N(10) atoms, remains bound to the N(5) atom. The resulting N(5)=C(11)H_2_ group is responsible next for the electrophilic attack on the pyrimidine ring C(5) of the substrate/analog [13]. Thus, with N^4^-OH-dCMP bound in the active site the imidazolidine ring opens at the N(5) site, in a striking contrast to the corresponding phenomenon observed with dUMP and FdUMP, the difference resulting apparently from the presence of the N^4^-OH residue. As a consequence, the distance between the cofactor N(10)-C and pyrimidine C(5) atoms is too large to allow the electrophilic attack (in the 6F6Z structure subunits A and B the corresponding distances are 4.55 Å and 4.39 Å, respectively), thus resulting in the apparent lack of meTHF methylene group transfer to the pyrimidine C(5) atom. The latter may be the reason why TS inhibition by N^4^-OH-dCMP, unlike by FdUMP (cf. PDB ID: 5FCT), does not lead to the formation of a covalently-bound ternary complex, as evidenced by the 4EZ8 and 5M4Z structures.

The one-carbon moiety in the 6F6Z structure is relatively near Phe219 and Asn220 residues (the distances between C(11) and phenyl ring centroid of Phe219, and between C(11) and Nδ of Asn220 amount to 4.42 Å and 3.49 Å, and 4.65 Å and 3.68 Å, in subunits A and B, respectively), the latter residue being responsible for the ability of TS to discriminate dUMP from dCMP [34]. However, the one-carbon group bound to the N(10) atom does not affect conformations of Phe219 and Asn220 residues that remain unaltered compared to their conformations in the 4EIN and 4EZ8 structures.

Considering potential differences between mammalian and nematode TS, Table 1 gathers distances between the O(4) atom of the N(4)-OH group and the Tyr129 and His190 sidechains (or their counterparts); the latter sidechains are chosen for remaining in close proximity to the N(4)-OH group and, at the same time, exhibiting the most noticeable differences between structures discussed herein. Of note, is that the distances between O(4) and the sidechains of Tyr129 and His190 are larger in the mTS structures (4EIN, 4EZ8, and 6F6Z) than in the nematode TS structures (4PSG and 5M4Z). Apart from that, no further recurring structural differences are apparent for any particular group of structures.

The intermediate step of the reaction captured in the 6F6Z structure provides an answer to the question as to why the one-carbon group is not transferred from meTHF to the pyrimidine ring C(5) atom, as is the case with FdUMP. However, the further fate of the one-carbon group, the exact mechanism causing the unusual imidazolidine ring opening, as well as the mechanism of N^4^-OH-dCMP pyrimidine ring reduction and formation of a covalent bond with the catalytic cysteine residue, are yet to be revealed.

## 4. Material and Methods

(6*RS*)-methylene-5,6,7,8-tetrahydrofolic acid was from Schircks Laboratories (Bauma, Switzerland). N^4^-OH-dCMP was synthesized as previously described [9], with the use of hydroxylamine and dCMP from Sigma-Aldrich (St. Louis, MO, USA) as substrates.

### 4.1. Enzyme Preparation

Mouse (mTS), *C. elegans* (*Ce*TS), and *T. spiralis* (*Tsp*TS) thymidylate synthase recombinant proteins were overexpressed and purified as previously described [22,35,36,37,38], with phosphatase inhibitors (50 mM NaF, 5 mM Na-pyrophosphate, 0.2 mM EGTA, 0.2 mM EDTA and 2 mM Na_3_VO_4_) present in the purification buffers. TS activity was measured either spectrophotometrically [39] or with the use of the tritium release assay [40]. Purified TS preparation was separated into phosphorylated and non-phosphorylated fractions using metal oxide/hydroxide affinity chromatography (MOAC) on Al(OH)_3_ beads [38,41]. Protein contents of TS preparations was determined spectrophotometrically at ʎ = 280 nm [42].

### 4.2. Spectrophotometric Monitoring of a Time-Dependent DHF Production

Ultraviolet absorption spectra, in the 240–400 nm range, were recorded and calculations of specific activity performed as previously described [23], but for the compositions of the control (enzyme + meTHF) and complete (enzyme + nucleotide inhibitor + meTHF) reaction mixtures, containing 5.0 µM meTHF and 5.0 µM N^4^-OH-dCMP and the reaction run with 0.32 µM mTS dimer at 15 °C or 0.40 µM mTS at 8 °C. DHF concentration increase was determined by comparing the absorption increase at l_obs_ = 340 nm (a wavelength allowing to monitor DHF with a relatively small interference from meTHF [43]) in the control and complete reactions. The corrections concerning the enzyme concentration (see [23]) in each sample allow comparing specific activities that are independent of the amount/concentration of the enzyme in buffer solution.

### 4.3. Crystallization and Data Collection

Purified TS non-phosphorylated fractions were used for crystallization. Each TS solution underwent buffer exchange to 5 mM Tris-HCl pH 7.5 with 5 mM DTT. Crystals were grown by the vapor diffusion method at a temperature of approximately 4–7 °C. Concentrations of proteins and ligands, as well as a composition of well solution used to obtain each crystal, are given in Table 2. In every case a drop of the protein–ligand solution was mixed at 1:1 volume ratio with the corresponding well solution. The structure of mTS-N^4^-OH-dCMP complex soaked with meTHF was obtained by soaking crystals of the initial complex with approximately 50 mM solution of meTHF for a few hours. The structure of *Ce*TS with N^4^-OH-dCMP was obtained by soaking crystals of *Ce*TS apoenzyme with 36 mM solution of N^4^-OH-dCMP for approximately 80 min. X-ray diffraction data were collected from single flash-frozen crystals at the BESSY synchrotron beamline 14.1 using X-rays of 0.918 Å wavelength, except for the *Ce*TS-N^4^-OH-dCMP complex crystal whose data were collected using the Nova high-flux-micro-focus sealed tube X-ray source at 1.541 Å. Approximately 15% (*v*/*v*) solutions of glycerol in well solution buffers were used as cryoprotectants.

### 4.4. Structure Determination and Refinement

Data were processed with Denzo and Scalepack [44], except for those collected from the *Ce*TS-N^4^-OH-dCMP crystal that were processed with CrysAlisPro (Agilent Technologies, Santa Clara, CA, USA) and SCALA [45]. Structures were determined by molecular replacement carried out with the Phaser from CCP4 package [46]. The structures from the Protein Data (PDB), including 4EIN and 4G9U (superseded by 5BY6), were chosen as the search models for structures of complexes of *Ce*TS-N^4^-OH-dCMP, *Tsp*TS-N^4^-OH-dCMP cocrystallized with meTHF, and mTS-N^4^-OH-dCMP soaked in meTHF. Repetitive cycles of model rebuilding/adjustments were carried out with Coot [47] and refinement using Refmac5.5 [48] from the CCP4 suite or phenix.refine [49] from the Phenix suite [50]. Correctness of the structures was evaluated using PROCHECK [51], MolProbity [52], POLYGON [53], and Sfcheck [54]. X-ray data and final model refinement parameters are given in Table 3. OMIT maps were generated with the phenix.composite_omit_map tool using the “simple” method [55].

## Figures and Tables

**Table 1:** Distances between selected atoms in the crystal structures of TS from various sources: 5M4Z, 4EZ8 [14], 6F6Z, 4EIN [14] and 4PSG.

Distance [Å] between:	4EZ8	5M4Z A	5M4Z B	6F6Z A	6F6Z B	4EIN A	4EIN B	4PSG A *	4PSG B *
Tyr129 hydroxyl and N(4)-OH oxygen atom	4.57	3.67	3.47	4.09	3.98	4.38	4.33	3.76	4.46
Centroid of imidazole ring of His190 and oxygen atom of N(4)-OH	4.54	4.20	4.13	4.17	4.20	4.35	4.29	4.57	5.03
Centroid of imidazole ring of His190 and centroid of phenyl ring of Tyr129	5.81	3.95	3.90	5.53	5.60	6.08	6.08	4.15	4.25

* Corresponding residues in *Ce*TS sequence: Tyr137, and His198.

**Table 2:** Crystallization conditions.

System	PDB ID	TS Concentration in the Protein-Ligand Solution [mg/mL]	Ligand Concentration in the Protein-Ligand Solution [mM]	Ligand Concentration Used for Crystal Soaking [mM]	Composition of the Well Solution ^c^
*Tsp*TS-N^4^-OH-dCMP cocrystallized with meTHF	5M4Z	20	N^4^-OH-dCMP: 7 meTHF: 10	---	0.1M NaF pH 7.1 19% (*w*/*v*) PEG 3350
mTS-N^4^-OH-dCMP ^a^ soaked with meTHF	6F6Z	20	N^4^-OH-dCMP: 6	meTHF: ≈50	0.1M MES pH 6.7 0.15M Mg(CH_3_COO)_2_ 14% (*w*/*v*) PEG 8000
*Ce*TS ^b^ soaked with N^4^-OH-dCMP	4PSG	24	----	N^4^-OH-dCMP: ≈36	0.1M MES pH 5.6 0.2M Mg(CH_3_COO)_2_ 15% (*w*/*v*) PEG 8000

^a^ Reference [14]; ^b^ Reference [18]; ^c^ Well solutions contained 0.02% (*w*/*v*) NaN_3_.

**Table 3:** X-ray data and final model refinement parameters.

	*Ce*TS Soaked with N^4^-OH-dCMP	*Tsp*TS Cocrystallized with N^4^-OH-dCMP and meTHF	mTS Cocrystallized with N^4^-OH-dCMP and Soaked with meTHF
PDB ID	4PSG	5M4Z	6F6Z
Crystal properties and data collection			
Matthews coefficient [Å^3^/Da] (solvent content [%])	3.17 (61.2)	2.07 (40.5)	3.37 (63.5)
Space group	P 1 2_1_ 1	P 1	C 1 2 1
Cell dimensions	a = 59.4 Å, b = 95.9 Å, c = 82.2 Å β = 103.8° α = γ = 90°	a = 51.3 Å, b = 52.8 Å, c = 63.9 Å α = 72.5°, β = 68.0°, γ = 61.2°	a = 160.4 Å, b = 89.2 Å, c = 66.6 Å β = 97.5° α = γ = 90°
Wavelength [Å]	1.54	0.918	0.918
Resolution [Å] ^a^	2.80–79.80 (2.80–2.95)	1.18–45.72 (1.18–1.21)	2.13–48.81 (2.13–2.26)
Unique reflections	22154	172936	51781
Completeness [%] ^a^	99.8 (99.9)	97.4 (89.0)	98.2 (98.35)
Redundancy	8.7	3.61	3.42
I/σ(I) ^a^	8.1 (5.53)	16.43 (1.76)	14.6 (1.11)
Refinement			
Resolution [Å]	2.80–25.11	1.18–28.09	2.13–47.82
Number of reflections	22128	172936	50161
R_work_ [%] ^a^	23.1 (33.5)	13.8 (28.1)	21.0 (37.2)
R_free_ [%] ^a^	26.7 (41.4)	15.1 (28.4)	24.3 (40.3)
Average B, all atoms [Å^2^]	16.6 ^b^	23.7	49.8
RMS deviations from ideal values for refined atoms			
RMS_bond_ [Å]	0.011	0.008	0.006
RMS_angle_ [°]	1.607	1.086	0.848
Non-hydrogen atoms used in refinement			
Protein atoms	4632	4676	4638
Solvent atoms	62	291	250
Heterogen atoms	42	125	108
Ramachandran’s plot assignments			
Favored	556 (96%)	569 (98%)	553 (97%)
Allowed	19 (3%)	9 (2%)	17 (3%)
Disallowed	2 (0%)	2 (0%)	2 (0%)

^a^ Values in parentheses refer to the highest resolution shell. ^b^ Considering notably low crystallographic B-factors for 4PSG, it is of note that (i) these factors represent both vibrational movement and static disorder, their combination rendering sometimes unexpected results and (ii) to achieve reliable B-factors, high-resolution structural data are required [56], unlike those obtained for 4PSG.

## Data Availability

The crystal structures of the parasitic nematode *Trichinella spiralis* thymidylate synthase cocrystallized with N^4^-OH-dCMP and N^5,10^-methylenetetra-hydrofolate, *Caenorhabditis elegans* thymidylate synthase crystal soaked with N^4^-OH-dCMP and of the binary mouse TS-N^4^-OH-dCMP complex crystal soaked with N^5,10^-methylene-tetrahydrofolate have been deposited under PDB accession codes 5M4Z, 4PSG and 6F6Z, respectively.

## Abbreviations

N^4^-OH-dCMP	N^4^-hydroxy-dCMP
meTHF	N^5,10^-methylenetetrahydrofolate
THF	tetrahydrofolate
DHF	dihydrofolate
TS	thymidylate synthase
m	mouse
*Tsp*	*Trichinella spiralis*
*Ce*	*Caenorhabditis elegans*
